# Wound healing complications in patients with and without systemic diseases following hallux valgus surgery

**DOI:** 10.1371/journal.pone.0197981

**Published:** 2018-06-01

**Authors:** Justyna Kromuszczyńska, Łukasz Kołodziej, Alina Jurewicz

**Affiliations:** Department of Orthopaedics, Traumatology and Musculoskeletal Oncology, Pomeranian Medical University, Szczecin, Poland; Medical University Innsbruck, AUSTRIA

## Abstract

There are many defined risk factors for wound healing. Comorbidities and their treatment are identified to be one of them. The aim of this study is to verify whether there are significant differences in wound healing between patients with and without systemic diseases, who underwent hallux valgus correction with Scarf osteotomy. A total of 155 consecutive patients were included into this prospective study. All of the patients underwent Scarf osteotomy for hallux valgus correction. In 60,6% of patients comorbidities were present, most often hypertension (57 patients, 36,8%), hypothyroidism (19 patients 12,3%) and diabetes (7 patients, 4,5%) occurred. Most of the patients were women (96,1%). During the study complication rate was noted. Patients underwent follow-up: 1,2, 3, 6 and 12 weeks and 6 months after the surgery. Preoperatively and during the last visit treatment results were assessed with AOFAS HMI scale. Scar assessment was performed by independent observer with VAS followed by patient scar assessment with VSS. In 30 patients complications were noted (19,4%). Surgical site infection was found in 6 patients (3,9%). In 13 patients (8,4%) partial wound dehiscence occurred, in 5 of them (3,2%) additional skin closure (Steri-Strips) was applied. Treatment results assessed with AOFAS HMI scale were good and very good in both healthy and comorbidity group, and the results improved significantly after surgical procedure. Scar assessment with VAS was on the average 1,5 pts. Average result in VSS was 2 pts. Results in both scales were rated as very good. No statistically significant differences were found in both healthy and comorbidity group in scar assessment. Based on the results of the study authors believe there are no significant differences between patients with and without comorbidities in aspects like: complication rate, surgery result and scar assessment as long as foot surgery is concerned.

## Introduction

Hallux valgus is the most common foot deformity in adults with the frequency of 2–4% in population [1–2]. This deformation 7–9 times more often occurs in women, depending on an author [3–5]. Its frequency increases with age, reaching the level of 35% in the population above 65 years [3, 6–7]. In addition, the incidence of chronic diseases is increasing with age. Comorbidities, such as diabetes, rheumatoid arthritis and other rheumatic diseases, smoking or obesity, play important part in wound healing and treatment result. The increasing role of cosmetic effect should be outlined, especially as the deformation concerns mostly women.

Wound healing complications are an important aspect to every surgeon. It is a particularly important issue to foot and ankle surgeons due to feet anatomical specificity: thick epidermis, thinner subcutaneous layer and a large number of exocrine glands as well as footwear and socks that cause temperature growth and higher CO2 and moisture levels (incubation). This contributes to high bacterial colonization [8–11] and automatically to greater risk of infection ranging from 1,44% to 11% [12–13].

There are many identified risk factors for wound healing complications. They can be divided into two basic groups: patients dependent and surgery dependent. Among surgery factors surgery technic, materials used for skin closure can be outlined. Patients factors include: age, BMI (body mass index), comorbidities and its pharmacotherapy, smoking, alcohol abuse, NSAIDs (non-steroidal anti-inflammatory drugs), nutrition and many others [14]. Systemic diseases that influence wound healing are: diabetes, rheumatoid arthritis and its treatment: use of steroids, disease-modifying anti-rheumatic drug (DMARD), and biological therapy, thyroxine hormone substitution [15–18].

The aim of this study was to establish type and rate of complications and to determine whether comorbidities increase the risk of complications and if they influence functional results and cosmetic effects of the surgery.

## Materials and methods

155 consecutive adult patients were included into this prospective study. Written consents were obtained. All the patients underwent surgery due to hallux valgus deformation with Scarf and Akin osteotomies from April 2015 and March 2016 in the Department of Orthopaedics, Traumatology and Musculoskeletal Oncology of Pomeranian Medical University in Szczecin. The study was approved by Bioethical Board of Pomeranian Medical University. Exclusion criteria were: revision surgery, local skin or nail infection, history of keloids in the past. One patients had bilateral procedures. Each foot was evaluated separately.

Patient demographics and medical comorbidities were recorded (age, gender, chronic diseases, medications, smoking status, BMI). 6 follow-ups were planned: 1,2, 3, 6 and 12 weeks and 6 months after the surgery. Check-ups took place in the Outpatient Clinic by an independent observer—the resident with experience in foot and ankle surgery. During each follow-up presence of any complication was noted. Preoperatively and during the last check-up treatment results with The American Orthopaedic Foot and Ankle Society Score: Hallux Metatarsophalangeal-Interphalangeal (AOFAS HMI) scale were assessed [19]. This is one of the most widely used, validated [20] scale to evaluate foot and ankle condition in version for hallux and the 1^st^ MTP (metatarsophalangeal) joint. It comprises 9 questions concerning 3 categories: pain (40 points), function (50 points), alignment (10 points). Total score is 100 points. The more points the better.

Scar appearance was evaluated during the last follow-up with The Vancouver Scar Scale (VSS, patient evaluation) and Visual Analogue Scale (VAS, independent observer evaluation). Both scales are subjective. VSS assesses 4 variables: vascularity, height/thickness, pliability and pigmentation. Scoring system ranges from 0 (excellent) to 13 points (poor). VSS is one of the most widely used scale for scar assessment and has been validated [21]. VAS evaluates scar in 4 dimensions (pigmentation, vascularity, acceptability and observer comfort) plus contour [22].

### Post-op protocol

All patients underwent routine postoperative protocol, which consisted of everyday dressing change and full weight bearing in a Barouk shoe for 5 weeks. During the hospitalization the patients were taught basic exercises increasing the range of motion in the 1^st^ MTP joint. Sutures were removed during the 2^nd^ or 3^rd^ follow-up depending on the healing state and the thread used.

### Demographics

149 of 155 patients were women (96,1%) with the mean age of 57 years (range 23–74). Mean BMI was 24,9 kg/m^2^ (range 17,3–33,1). S1 Table presents the percentage of patients divided into 3 groups according to their BMI.

Over 60% of patients had a chronic disease (S2 Table). Specific comorbidities are shown in the S3 Table 28,4% patients chronically used medications (S4 Table). Drugs that patients were particularly asked about were: disease-modifying anti-rheumatic drug (DMARD), steroids, biological therapy, hormonal substitution and insulin intake (S5 Table). Only 14,8% of patients were smokers.

## Results

Total complications rate was 19,4% (30 patients in total, 17 with comorbidities and 13 without) (S6 and S7 Tables). 6 patients (3,9%) suffered from superficial infection (SSI—surgical site infection) (5 patient with chronic diseases and 1 without), that was treated with the course of oral empirical antibiotics (S8 Table).

Partial wound dehiscence occurred in 13 patients (8,4%, 5 patients with comorbidities and 8 without) (S9 Table). Only in five (3,2%) cases Steri-Strips were applied during the 1^st^ postoperative visit. This complication was noted when the wound was wider than 3 mm, none of the partial dehiscence exceeded 6 mm. In 14 patients (9%, 10 with chronic disease and 4 without) allergic reaction was observed (S10 Table).

There were no statistically significant differences in the frequency of complications among healthy patients and those with chronic conditions. No studied risk factor proved to be statistically significant (S11 Table).

The average AOFAS HMI scale results were 88,8 pts for patients without comorbidities and 87,4 pts for the other group (p>0,01). The average improvement after the surgery was 32,4 pts for healthy patients and 33,2 pts for comorbidities group, in both groups the improvement was significant (p<0,01).

VAS score in 56 patients (36,1%) were excellent (0 pts). 63 patients (40,7%) were assessed with 1–2 pts. 3 or more pts got 36 patients (23,2%). In VSS 33 patients (21,3%) scored 0 pts (excellent). 1 to 3 points got 93 patients (60%), 4 or more 29 patients (18,7%). There were no significant differences between comorbidity and healthy group (S12 Table).

## Discussion

Most of the available studies concerning discussed subject apply to surgery in general, there is only a few papers referring to foot and ankle surgery. Factors influencing wound healing have been a concern for surgeons for many years [23–24]. Many of them are identified. They can be categorized into local and systemic factors.

Among systemic factors *Guo and DiPietro* mentioned age, sex hormones in aged individuals, stress, diabetes and medications such as glucocorticosteroids (GCS), non-steroidal anti-inflammatory drugs (NSAIDs), chemotherapeutic drugs, obesity, alcohol consumption, smoking and nutrition [15]. In present study no statistically significant risk factors were identified.

*Momeni et al*. pointed obesity as a significant risk factor (p<0,05) of impaired wound healing after abdominoplasty and excluded smoking and previous abdominal surgery as potential ones. Complication rate in this study was 40,3% [25]. Similarly to present study, *Momeni et al* did not found a correlation between hypertension and increased risk of postoperative complication. Authors of present study noted complication rate at the level of 19,4% and excluded obesity as a risk factor of impaired wound healing. However, authors found smoking increases the risk of allergic reaction (OR = 3,8; 95% CI 1,14–12,6; p<0,05). Allergic reaction was present in 21,74% patients, who smoked and 6,82% of non-smokers.

*Kline et al*. found that overall complications incidence after operative treatment of pilon fracture occur more often in diabetic patients (71% to 35%, p = 0,011). Diabetes significantly increased the risk of surgical site infection (p<0,001). Wound complication was 7% in both diabetic group and control group [18]. Authors of present study noted that overall odds ratio (OR) for complications in diabetic patients was 1,71 (95% CI 0,32 to 9,3, p = 0,532).

*Grunfeld et al*. found that thyroxine supplementation is associated with higher risk of wound dehiscence in foot and ankle surgery. Wound dehiscence was observed in 10.8% healthy patients compared to 36.2% of patients receiving thyroxine (adjusted OR = 3.7, 95% CI 1.3 to 11.4), p = 0.01) [16]. In the present study authors found no statistically significant correlation between increased risk of wound dehiscence and thyroxine supplementation (OR = 1,91, 95% CI 0,95 to 4,89), p = 0,178).

In a paper of *Helal et al*. wound dehiscence was reported in 12% of patients after metatarsal osteotomies [26]. In presented study wound dehiscence was noted in 8,4%, which is consistent with the studies mentioned. Wound dehiscence was not increased either in patients with systemic diseases nor with chronic drug supplementation (p>0,01).

Surgical site infection (SSI) remains the most important complication in elective orthopedic surgery in rheumatoid arthritis (RA), with an incidence ranging from 2% to 15% [27–29]. *Den Broeder et al* noted 9% infection rate in patients with rheumatoid arthritis, who underwent foot and ankle surgery (OR 3,2, 95% CI 1,6–6,5, p = ,001). They established that prior skin or wound infection (OR 13.8, 95% CI 5.2–36.7) were associated with increased risk of SSI [17]. Authors of present paper found no correlation between RA, DMARD or steroids treatment (GCS) and increased risk of SSI (RA—OR 0,66, 95% CI 0,01–2,63, p = 0,221; DMARD—OR 0,83, 95% CI 0,09–7,36, p = 0,865; GCS—OR 1,28, 95% CI 0,33–4,96, p = 0,723).

*Gosain et al* similarly to presented results did not qualify the age as a factor of impaired wound healing. In their study wound healing in aged individuals is altered. Healing may last longer, but the final results are qualitatively similar to young subjects [30]. We observed that age increases the risk of complication frequency (OR 0,98, 95% CI 0,95–1,01, p = 0,130), however the correlation is not statistically significant.

*Ahn et al* outlined that smoking is harmful to both chronic and acute wounds because of tissue hypoxia [31]. Our data shows that smoking increases the risk of complication with the OR = 2,07, 95% CI 0,77–5,61, p = 0,151.

The limitations of the current study include considering not a single chronic disease, but many of them. The discrepancies between present paper and studies mentioned in the discussion may be caused by different patients sample included into each study. Some of the quoted papers refer to trauma patients, whereas procedures performed in present paper (Scarf and Akin osteotomies) are elective.

Authors believe that outlined outcomes are a wide presentation of wound healing problems after hallux valgus correction. However, further studies should be performed to provide information on the possible cause of wound complication and negative cosmetic effect of a scar.

## Conclusions

Study proves that there are no statistically significant differences in the results of surgical treatment of hallux valgus deformity between patients with and without comorbidities as far as complication rate, functional and esthetic outcomes are concerned. No statistically significant risk factor of wound complication was identified.

## Supporting information

S1 Table Patient groups concerning BMI. (PDF)

S2 Table Percentage of patients with and without chronic disease. (PDF)

S3 Table Comorbidities and its number in studied population. CKD—chronic kidney disease, Hep. C—hepatitis C, GERD—gastro-esophageal reflux disease, CLL—chronic lymphocytic leukemia, RA—rheumatoid arthritis, AF—atrial fibrillation, IHD—ischemic heart disease, COPD—chronic obturative pulmonary disease, HA—arterial hypertension.
(PDF)

S4 Table Proportion of patients with the chronic use of medications. (PDF)

S5 Table Specific drugs used by patients with systemic diseases. (PDF)

S6 Table Frequency and types of complications. SSI—surgical site infection.
(PDF)

S7 Table Complications frequency in patients with and without chronic diseases. (PDF)

S8 Table SSI frequency in patients with and without chronic diseases. (PDF)

S9 Table Wound dehiscence frequency in patients with and without chronic diseases. (PDF)

S10 Table Allergic reaction frequency in patients with and without chronic diseases. (PDF)

S11 Table The impact of the different factors on complication occurrence. BMI—body mass index, COPD—chronic obturative pulmonary disease, IHD—ischemic heart disease, RA—rheumatoid arthritis, GERD—gastro-esophageal reflux disease, DMARD—disease modifying antirheumatic drugs, GCS—glucocorticosteroids. No risk factor proved to be statistically significant (p>0,05).
(PDF)

S12 Table Correlation between comorbidities and VSS results. NVSS 0—patients scored 0 pts in VSS, NVSS1—patients scored 1—3 pts. NVSS 2—patients scored 4 or more pts. No statistically significant differences were found between healthy and comorbidities group.
(PDF)
