# Supplementary figures and images for: Wound healing complications in patients with and without systemic diseases following hallux valgus surgery
Source: PLoS One. 2018 Jun 1;13(6):e0197981. doi: 10.1371/journal.pone.0197981 (PMC5983514; doi:10.1371/journal.pone.0197981)

**Table 1. Patient groups concerning BMI.**

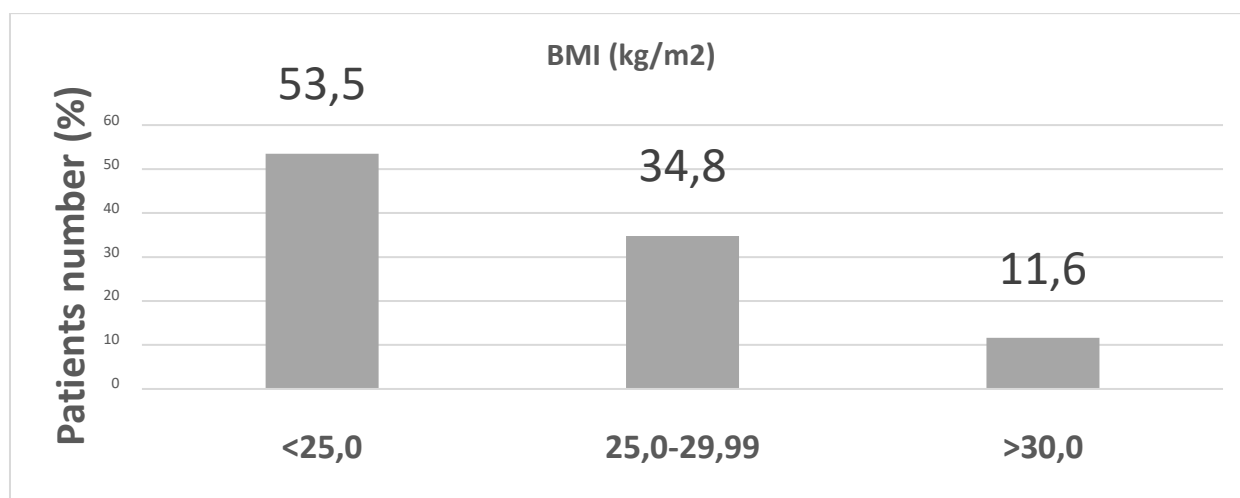

Supplement: S1 Table — (PDF) [file pone.0197981.s001.pdf]

**Table 2. Percentage of patients with and without chronic disease.**

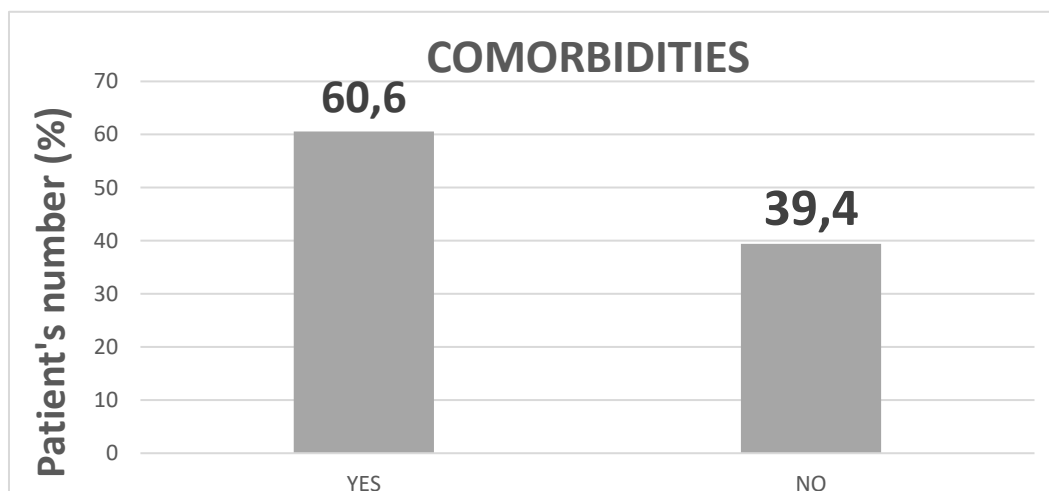

Supplement: S2 Table — (PDF) [file pone.0197981.s002.pdf]

**Table 4. Proportion of patients with the chronic use of medications.**

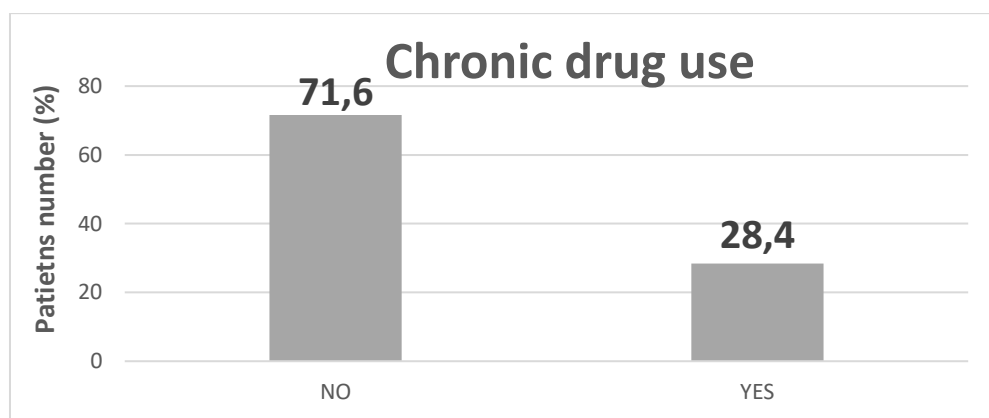

Supplement: S4 Table — (PDF) [file pone.0197981.s004.pdf]

**Table 5. Specific drugs used by patients with systemic diseases.**

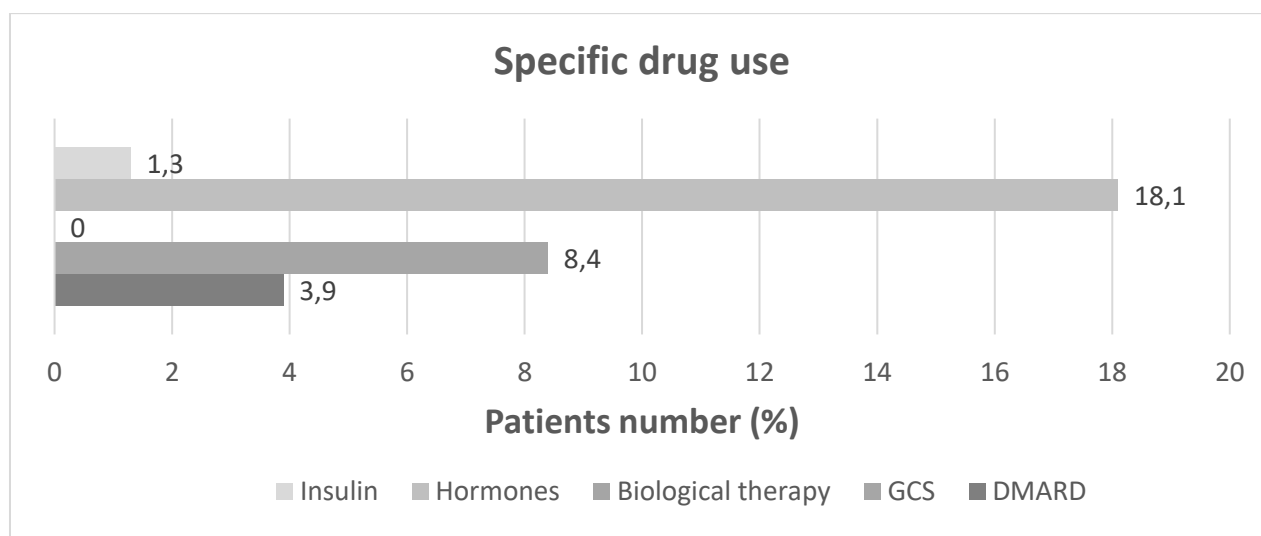

Supplement: S5 Table — (PDF) [file pone.0197981.s005.pdf]

**Table 6. Frequency and types of complications.**

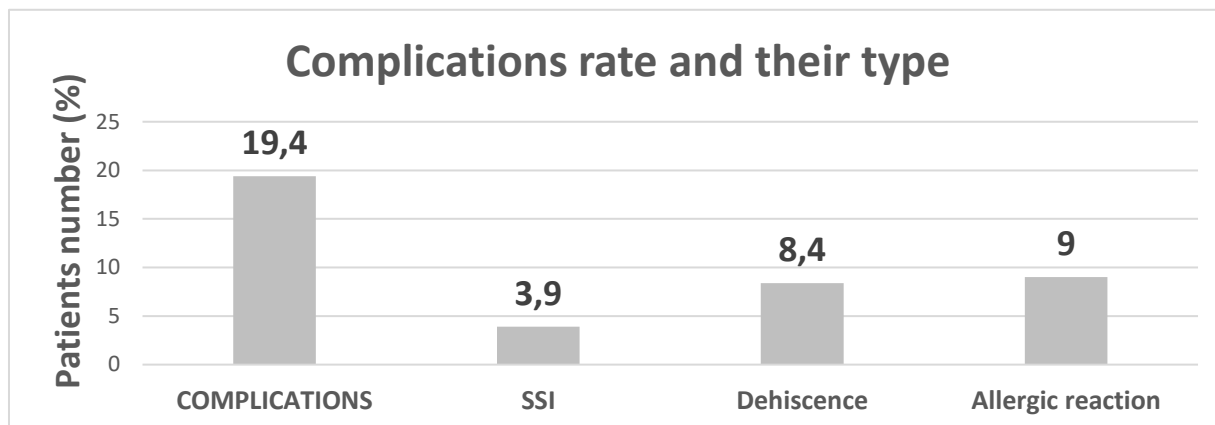

SSI – surgical site infection.

Supplement: S6 Table — SSI—surgical site infection. (PDF) [file pone.0197981.s006.pdf]
