# Supplementary material for: Wound healing complications in patients with and without systemic diseases following hallux valgus surgery
Source: PLoS One. 2018 Jun 1;13(6):e0197981. doi: 10.1371/journal.pone.0197981 (PMC5983514; doi:10.1371/journal.pone.0197981)
Supplement: S3 Table — CKD—chronic kidney disease, Hep. C—hepatitis C, GERD—gastro-esophageal reflux disease, CLL—chronic lymphocytic leukemia, RA—rheumatoid arthritis, AF—atrial fibrillation, IHD—ischemic heart disease, COPD—chronic obturative pulmonary disease, HA—arterial hypertension. (PDF) [file pone.0197981.s003.pdf]

Table 3. Comorbidities and its number in studied population.

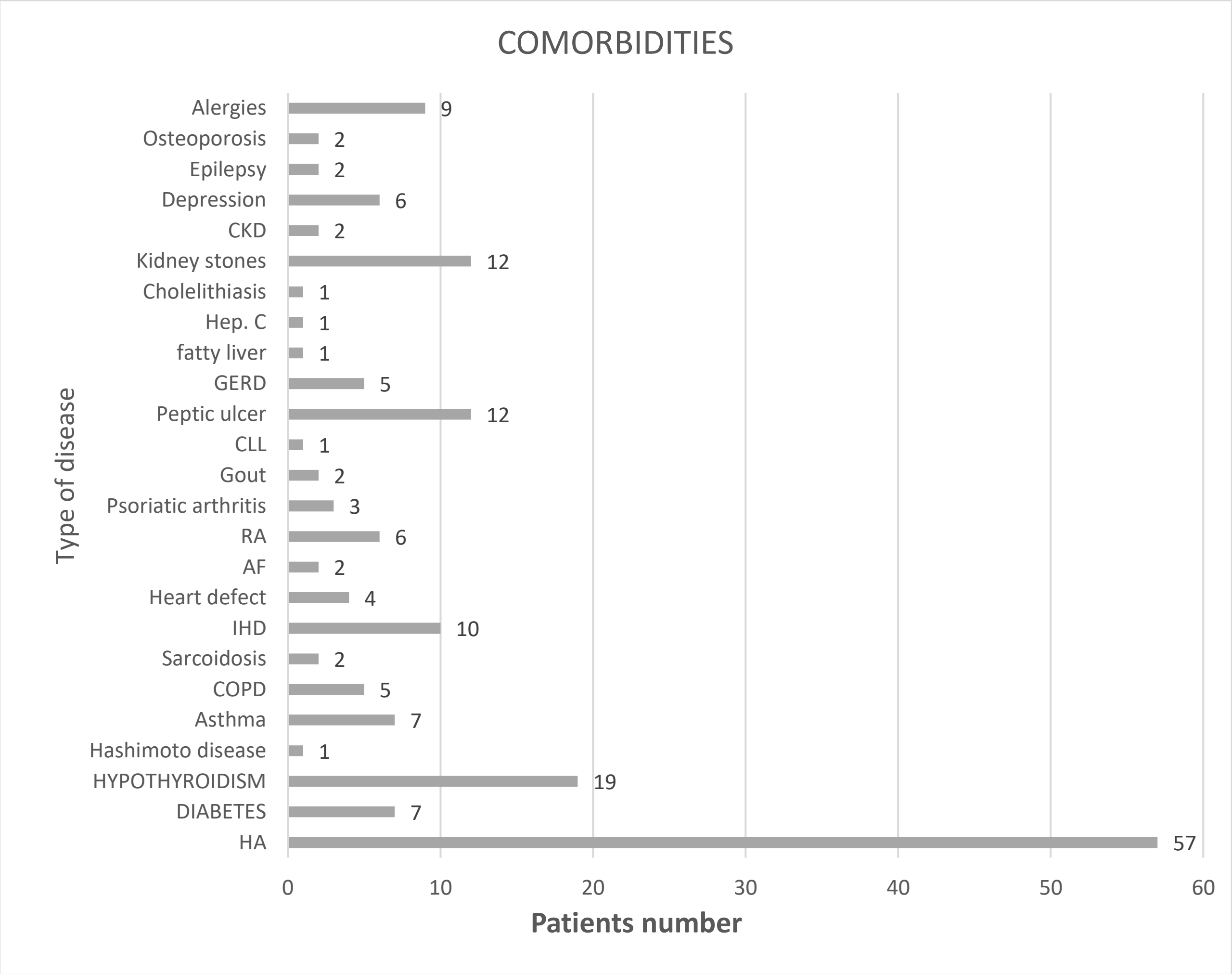

CKD – chronic kidney disease, Hep. C – hepatitis C, GERD – gastro-esophageal reflux disease, CLL – chronic lymphocytic leukemia, RA - rheumatoid arthritis, AF – atrial fibrillation, IHD – ischemic heart disease, COPD – chronic obturative pulmonary disease, HA – arterial hypertension.
