# Supplementary material for: Wound healing complications in patients with and without systemic diseases following hallux valgus surgery
Source: PLoS One. 2018 Jun 1;13(6):e0197981. doi: 10.1371/journal.pone.0197981 (PMC5983514; doi:10.1371/journal.pone.0197981)
Supplement: S7 Table — (PDF) [file pone.0197981.s007.pdf]

**Table 7. Complications frequency in patients with and without chronic diseases.**

| | COMPLICATIONS | | | | |
|---|---|---|---|---|---|
| **COMORBIDITIES** | **NO** | | **YES** | | Total |
| **NO** | 48 | 78,69% | 13 | 21,31% | 61 |
| **YES** | 77 | 81,91% | 17 | 18,09% | 94 |
| **Total** | 125 | | 30 | | 155 |
| Chi^2 Pearsona | 0,25 | | df=1 | | p=,61940 |
| R rang Spearman | -0,04 | | t=-,4939 | | p=,62210 |
