# Supplementary material for: Wound healing complications in patients with and without systemic diseases following hallux valgus surgery
Source: PLoS One. 2018 Jun 1;13(6):e0197981. doi: 10.1371/journal.pone.0197981 (PMC5983514; doi:10.1371/journal.pone.0197981)
Supplement: S8 Table — (PDF) [file pone.0197981.s008.pdf]

Table 8. SSI frequency in patients with and without chronic diseases.

	SSI				
**COMORBIDITIES**	**NO**		**YES**		Total
**NO**	60	98,36%	1	1,64%	61
**YES**	89	94,68%	5	5,32%	94
**Total**	149		6		155
Chi^2 Pearsona	1,35		df=1		p=,24595
R rang Spearman	0,09		t=1,1578		p=,24876
