# Supplementary material for: Wound healing complications in patients with and without systemic diseases following hallux valgus surgery
Source: PLoS One. 2018 Jun 1;13(6):e0197981. doi: 10.1371/journal.pone.0197981 (PMC5983514; doi:10.1371/journal.pone.0197981)
Supplement: S9 Table — (PDF) [file pone.0197981.s009.pdf]

Table 9. Wound dehiscence frequency in patients with and without chronic diseases.

	**WOUND DEHISCENCE**				
**COMORBIDITIES**	**NO**		**NO**		Total
**NO**	53	86,89%	8	13,11%	61
**YES**	89	94,68%	5	5,32%	94
**Total**	142		13		155
Chi^2 Pearsona	2,93		df=1		p=,08717
R rang Spearman	-0,14		t=-1,716		p=,08824
