# Supplementary material for: Wound healing complications in patients with and without systemic diseases following hallux valgus surgery
Source: PLoS One. 2018 Jun 1;13(6):e0197981. doi: 10.1371/journal.pone.0197981 (PMC5983514; doi:10.1371/journal.pone.0197981)
Supplement: S10 Table — (PDF) [file pone.0197981.s010.pdf]

Table 10. Allergic reaction frequency in patients with and without chronic diseases.

	ALLERGIC REACTION				
**COMORBIDITIES**	**NO**		**YES**		Total
**NO**	57	93,44%	4	6,56%	61
**YES**	84	89,36%	10	10,64%	94
**Total**	141		14		155
Chi^2 Pearsona	0,75		df=1		p=,38653
R rang Spearman	0,07		t=,86241		p=,38981
