# Supplementary material for: Wound healing complications in patients with and without systemic diseases following hallux valgus surgery
Source: PLoS One. 2018 Jun 1;13(6):e0197981. doi: 10.1371/journal.pone.0197981 (PMC5983514; doi:10.1371/journal.pone.0197981)
Supplement: S11 Table — BMI—body mass index, COPD—chronic obturative pulmonary disease, IHD—ischemic heart disease, RA—rheumatoid arthritis, GERD—gastro-esophageal reflux disease, DMARD—disease modifying antirheumatic drugs, GCS—glucocorticosteroids. No risk factor proved to be statistically significant (p>0,05). (PDF) [file pone.0197981.s011.pdf]

Table 11. The impact of the different factors on complication occurrence.

RISK FACTOR	**OR [95% CI]**	**p**
Age	0,98 0,95 1,01	0,130
Sex	0,83 0,09 7,36	0,865
BMI	0,93 0,83 1,04	0,185
ANY comorbidities	0,82 0,36 1,83	0,620
hypertension	1,19 0,52 2,68	0,683
diabetes	1,71 0,32 9,30	0,532
hypothyroidism	0,76 0,21 2,79	0,675
Asthma	0,56 0,01 2,23	0,185
COPD	1,04 0,11 9,69	0,970
IHD	0,44 0,05 3,65	0,450
heart deffect	4,39 0,59 32,54	0,147
RA	0,66 0,01 2,63	0,221
psoriatic arthritis	2,12 0,19 24,19	0,545
ulcer disease	0,82 0,17 3,96	0,806
GERD	1,04 0,11 9,69	0,970
allergy	0,43 0,01 1,70	0,130
medications	1,34 0,57 3,15	0,504
DMARD	0,83 0,09 7,36	0,865
GCS	1,28 0,33 4,96	0,723
hormones	1,91 0,75 4,89	0,178
smoking	2,07 0,77 5,61	0,151

BMI – body mass index, COPD – chronic obturative pulmonary disease, IHD – ischemic heart disease, RA - rheumatoid arthritis, GERD – gastro-esophageal reflux disease, DMARD - disease modifying antirheumatic drugs, GCS – glucocorticosteroids. No risk factor proved to be statistically significant (p>0,05).
