# Supplementary material for: Wound healing complications in patients with and without systemic diseases following hallux valgus surgery
Source: PLoS One. 2018 Jun 1;13(6):e0197981. doi: 10.1371/journal.pone.0197981 (PMC5983514; doi:10.1371/journal.pone.0197981)
Supplement: S12 Table — NVSS 0—patients scored 0 pts in VSS, NVSS1—patients scored 1—3 pts. NVSS 2—patients scored 4 or more pts. No statistically significant differences were found between healthy and comorbidities group. (PDF) [file pone.0197981.s012.pdf]

Table 12. Correlation between comorbidities and VSS results.

	NVSS		NVSS		NVSS		
COMORBIDITIES	0		1		2		Total
No	16	26,23%	33	54,10%	12	19,67%	61
Yes	17	18,09%	60	63,83%	17	18,09%	94
Total	33		93		29		155
Chi^2 Pearson	1,79		df=2		p=,40938		
R rang Spearman	0,05		t=,64248		p=,52153		

NVSS 0 – patients scored 0 pts in VSS, NVSS1 – patients scored 1-3 pts. NVSS 2 – patients scored 4 or more pts. No statistically significant differences were found between healthy and comorbidities group.
